# Supplementary figures and images for: Myocardial infarction affects Cx43 content of extracellular vesicles secreted by cardiomyocytes
Source: Life Sci Alliance. 2020 Oct 23;3(12):e202000821. doi: 10.26508/lsa.202000821 (PMC7652393; doi:10.26508/lsa.202000821)

**Figure1A**

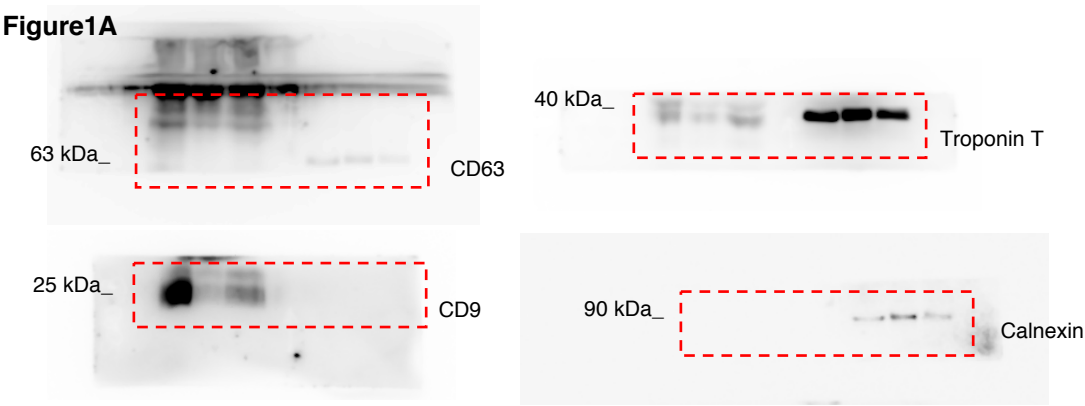

**Figure1D**

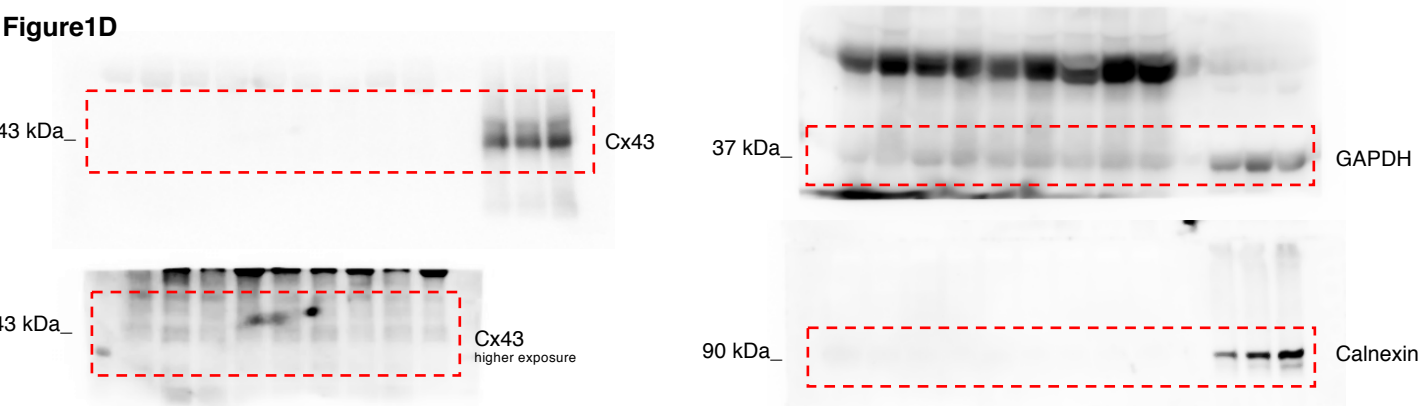

**Figure1E**

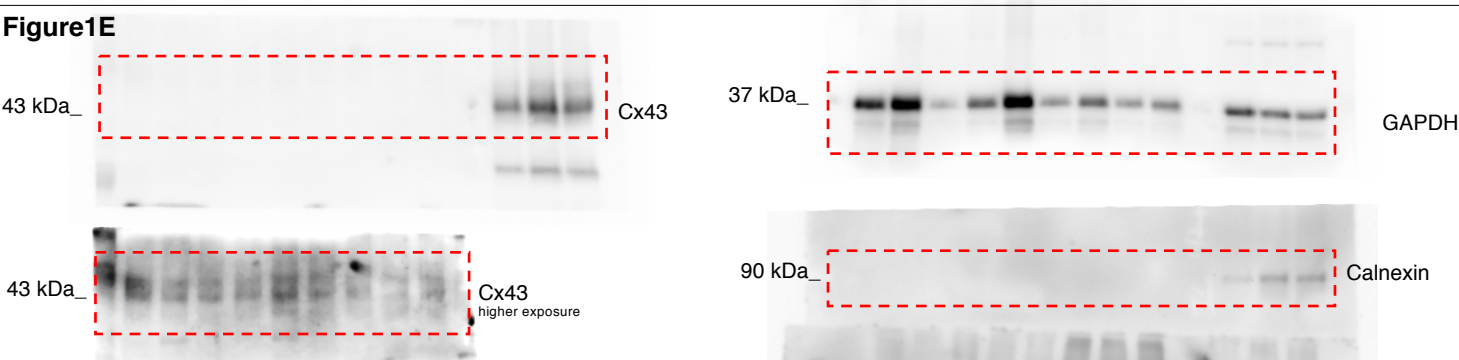

Supplement: Supplementary file 1 [file LSA-2020-00821_SdataF1.pdf]

**Figure2A**

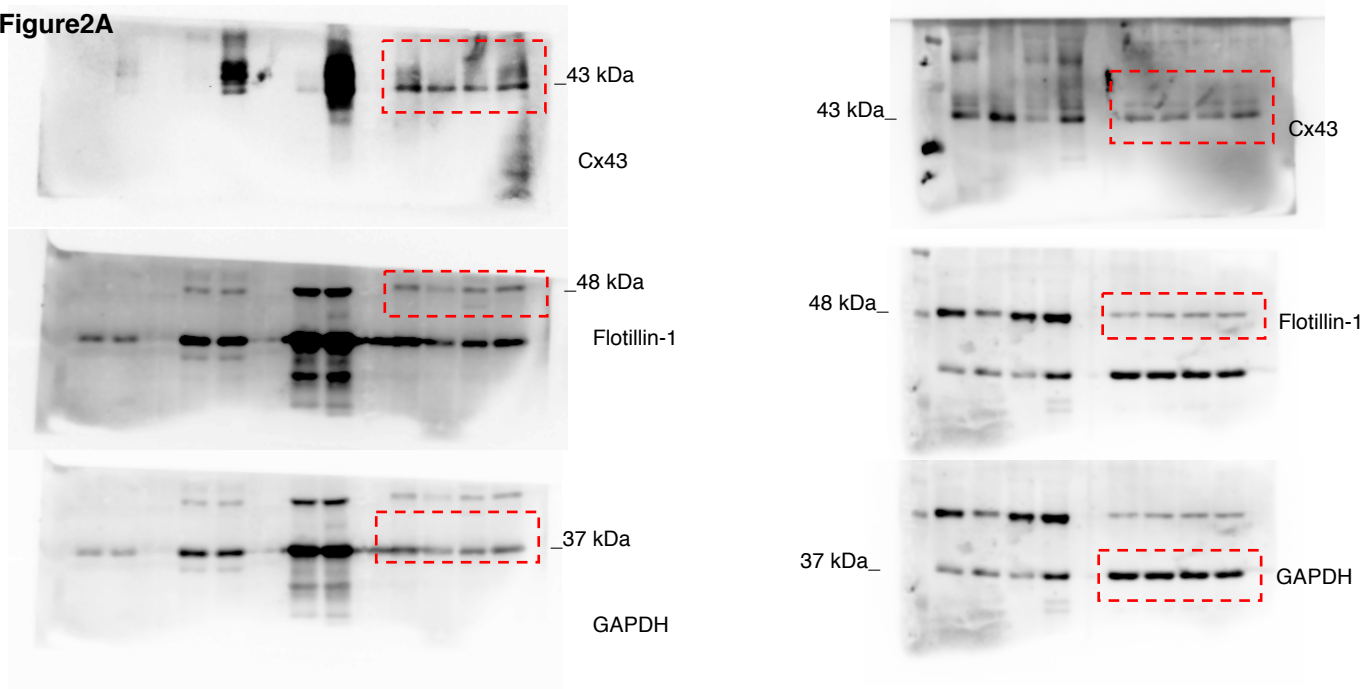

**Figure2B**

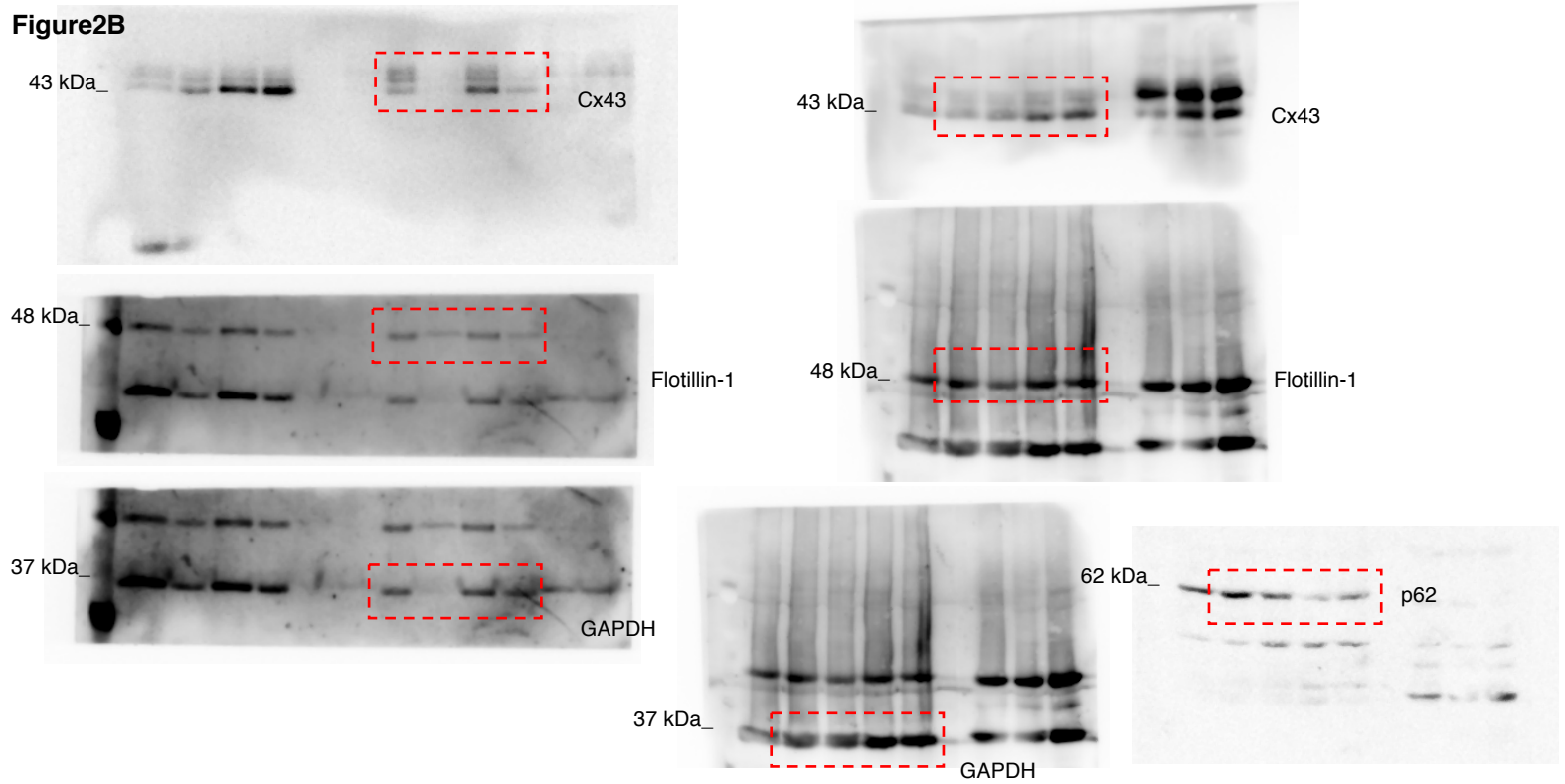

Supplement: Supplementary file 2 [file LSA-2020-00821_SdataF2.pdf]

**Figure3A**

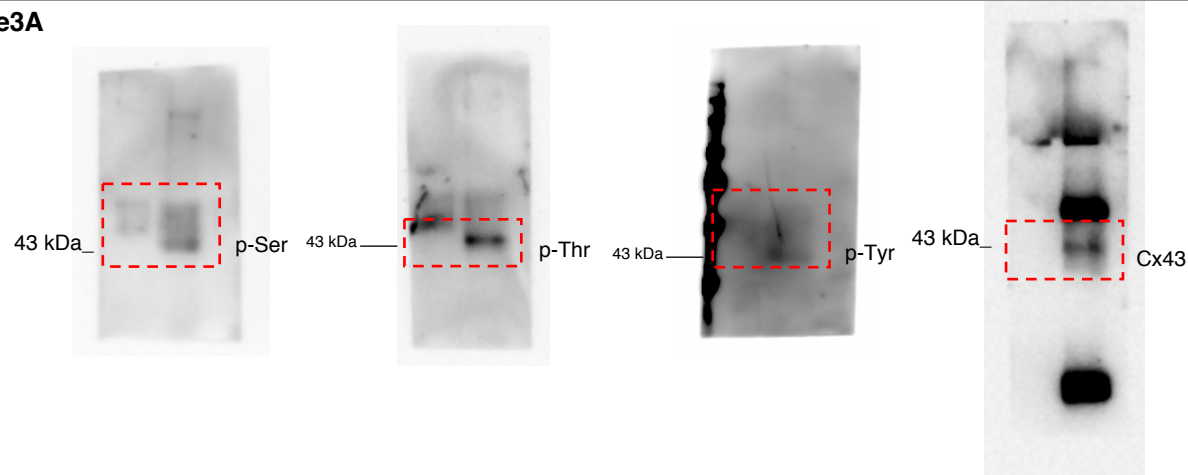

**Figure3B**

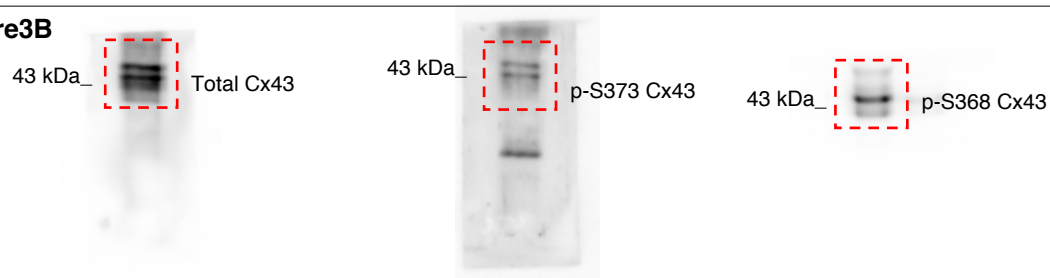

**Figure3C**

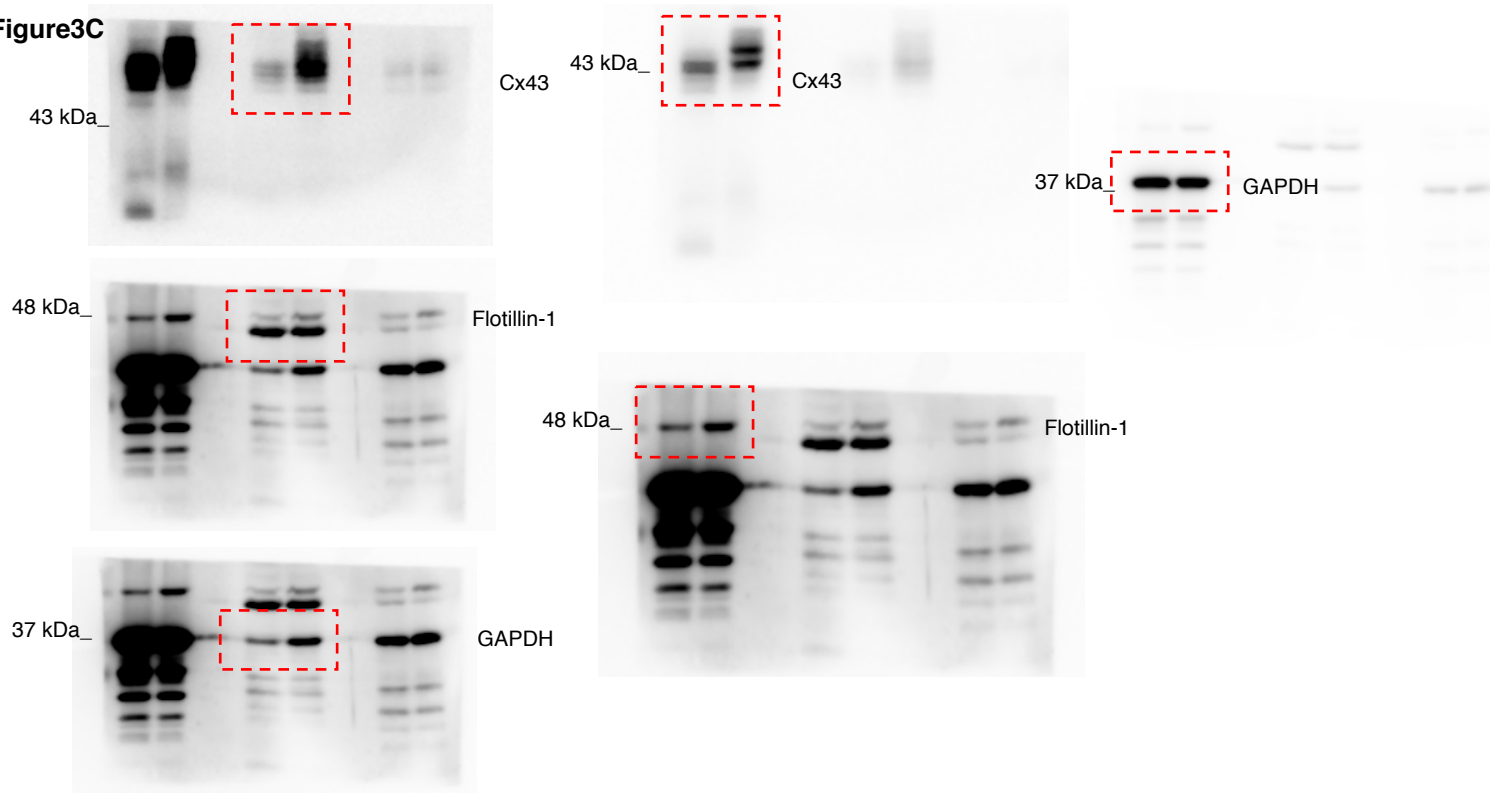

**Figure3D**

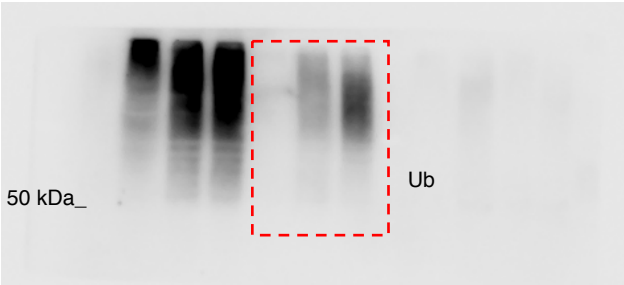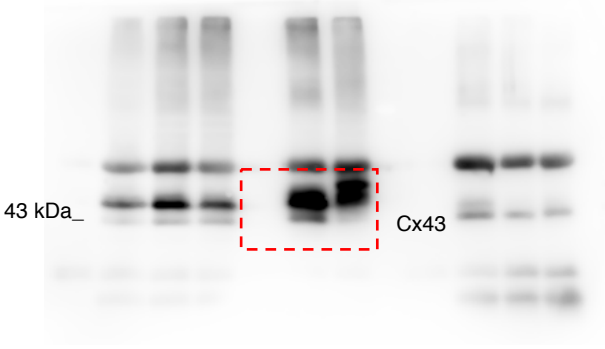

**Figure3E**

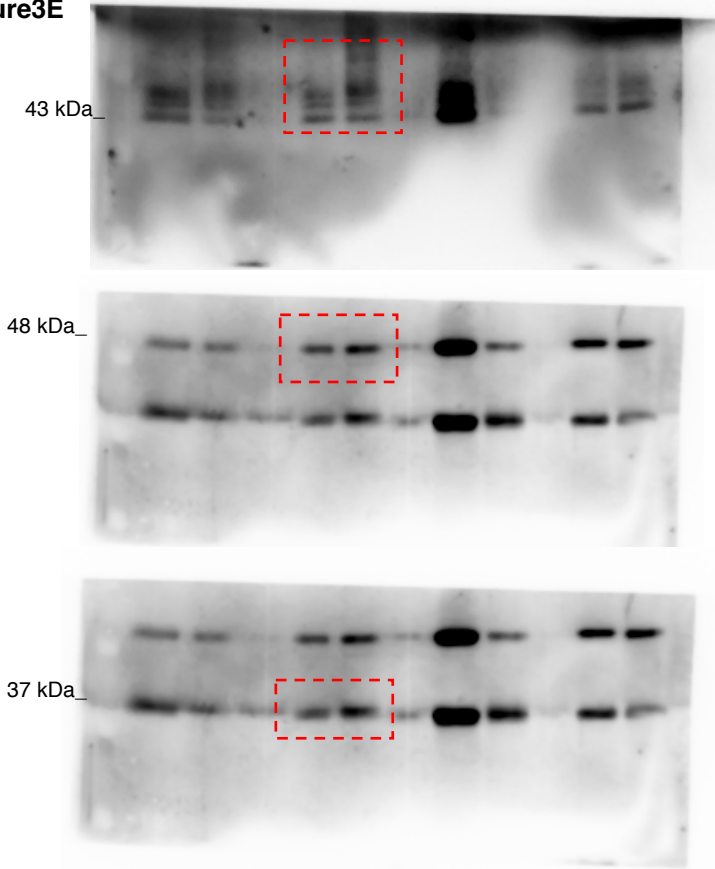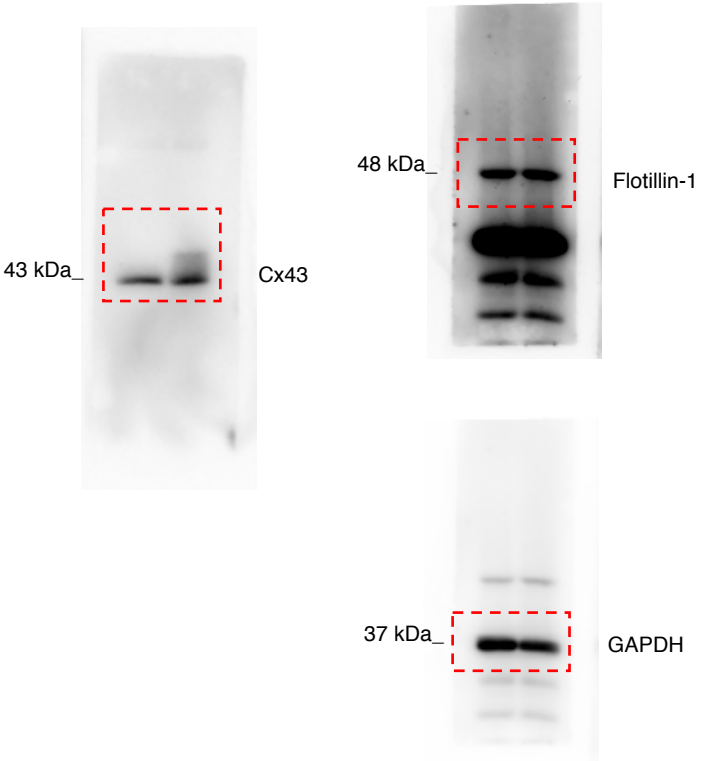

**Figure3F**

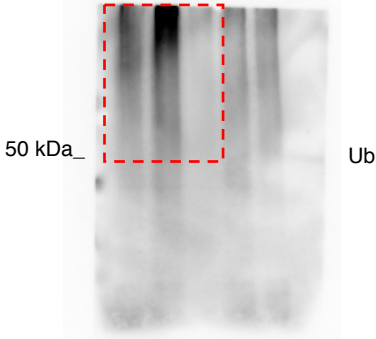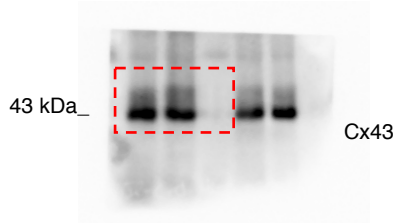

**Figure3G**

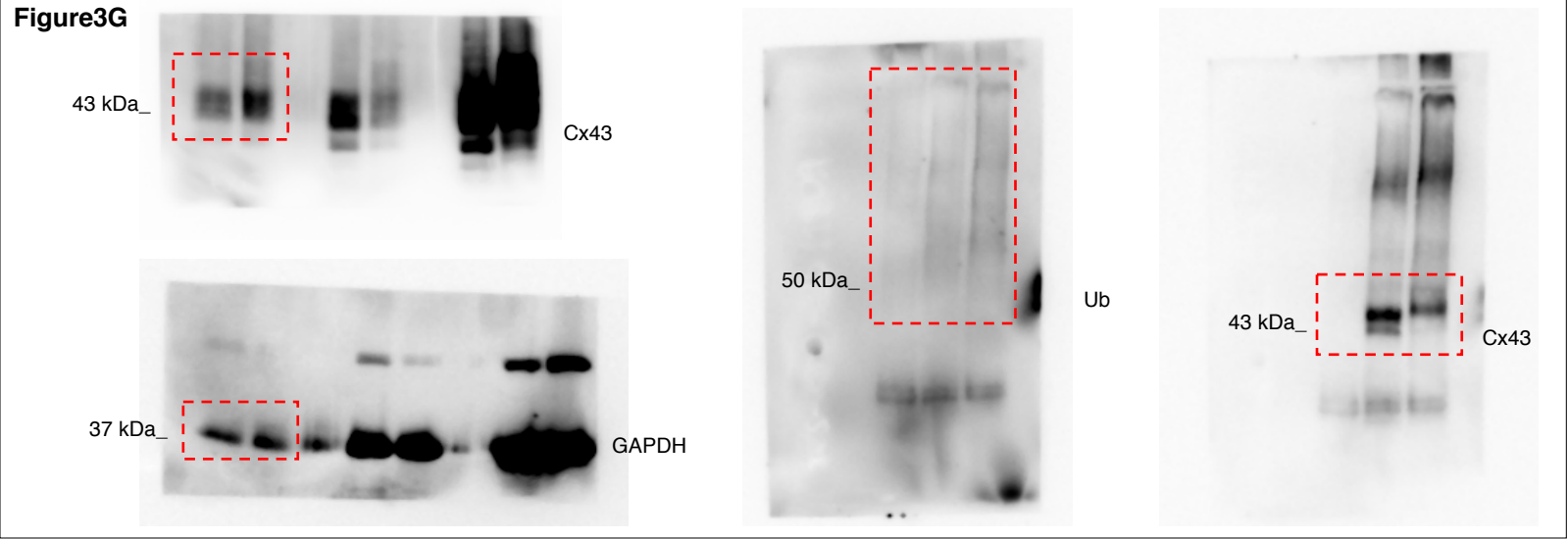

**Figure3H**

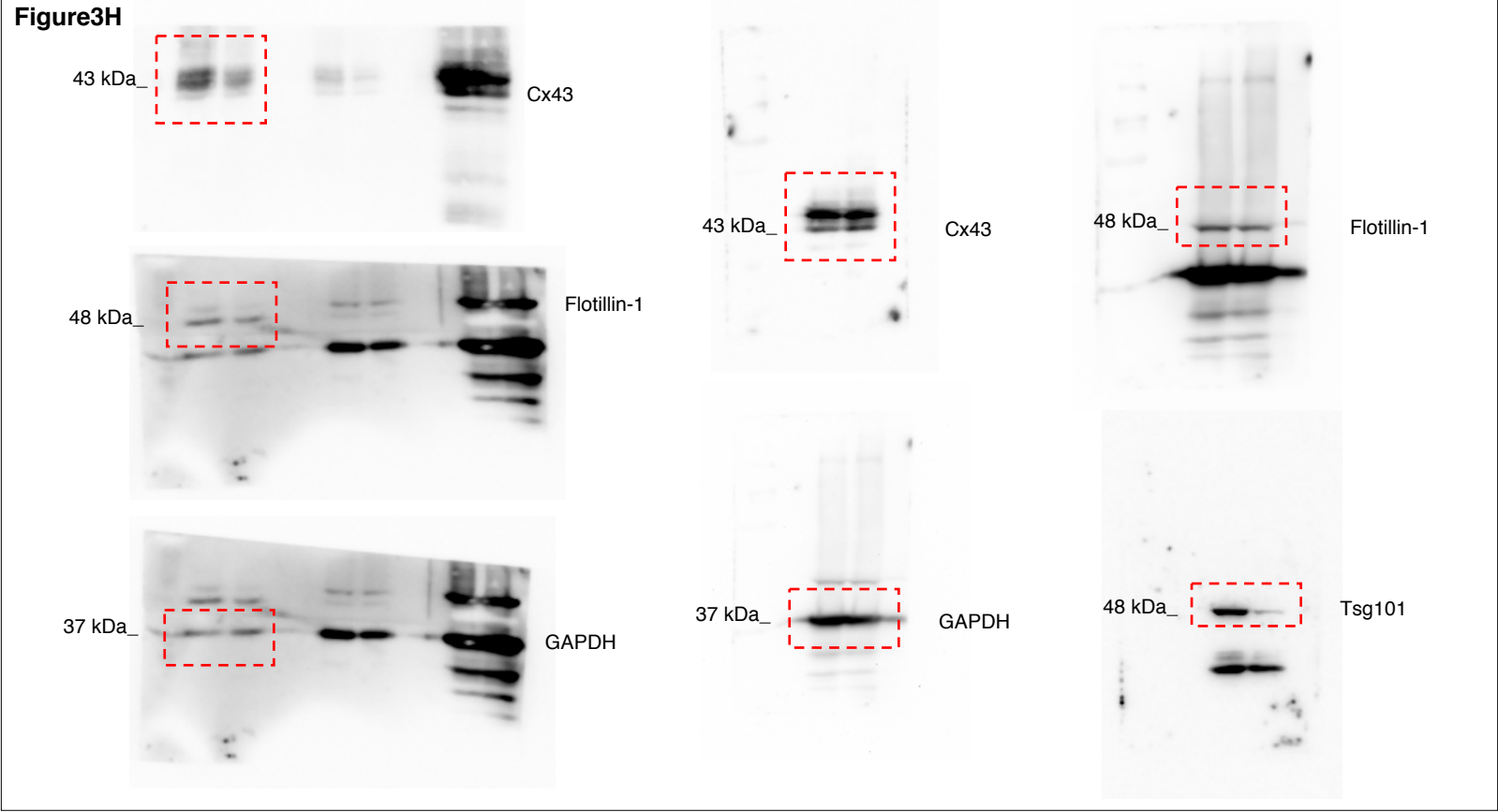

Supplement: Supplementary file 3 [file LSA-2020-00821_SdataF3.pdf]

**Figure4A**

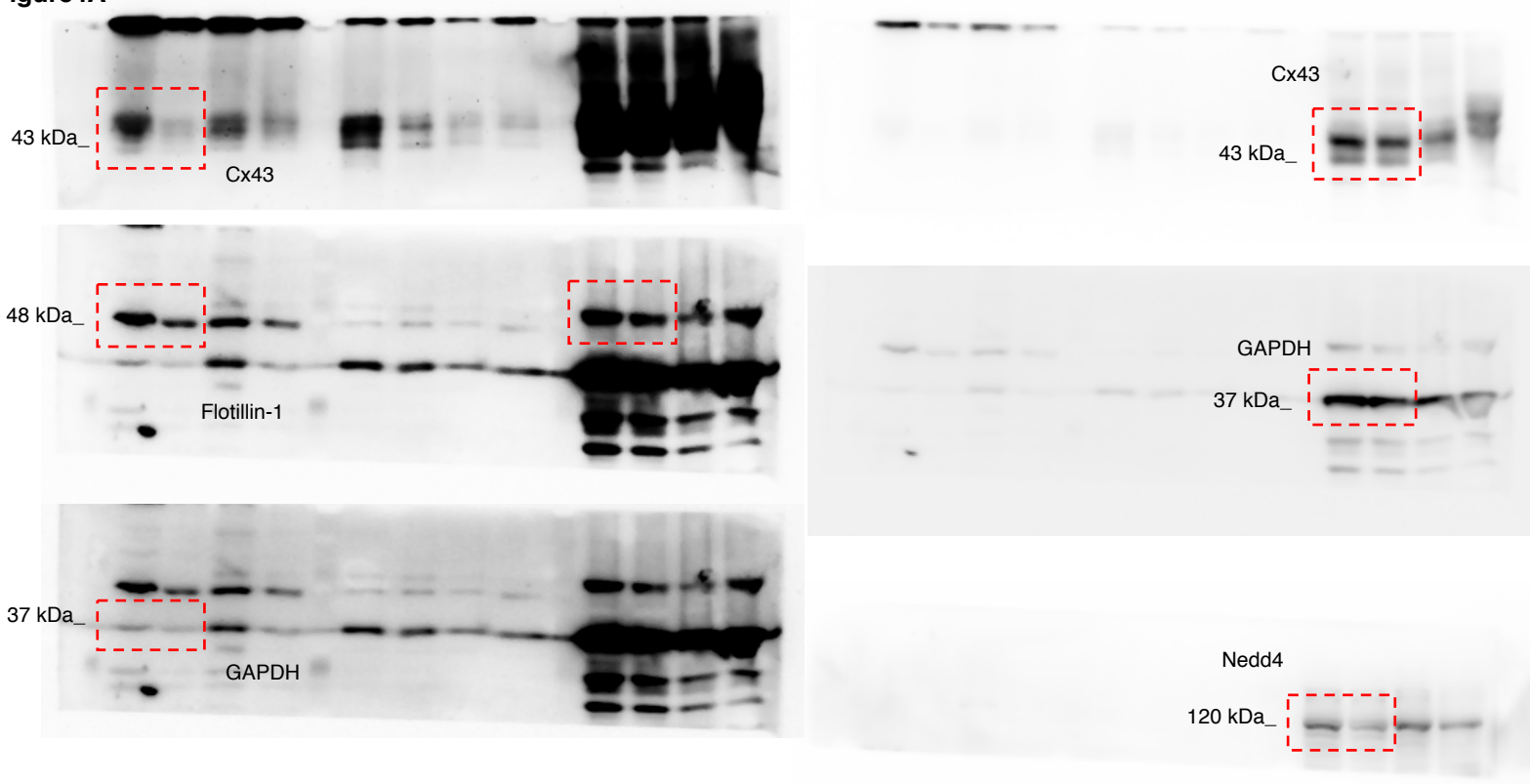

**Figure4B**

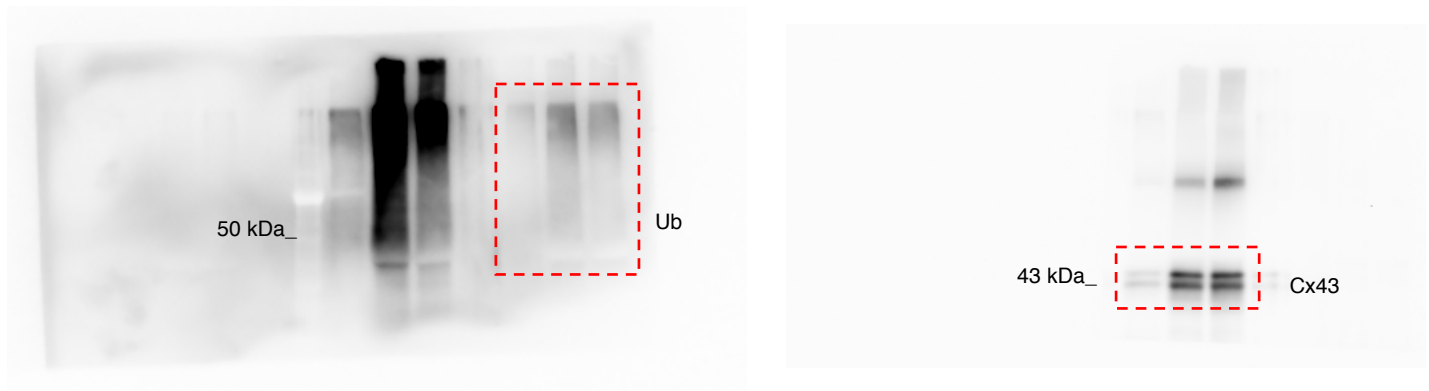

**Figure4C**

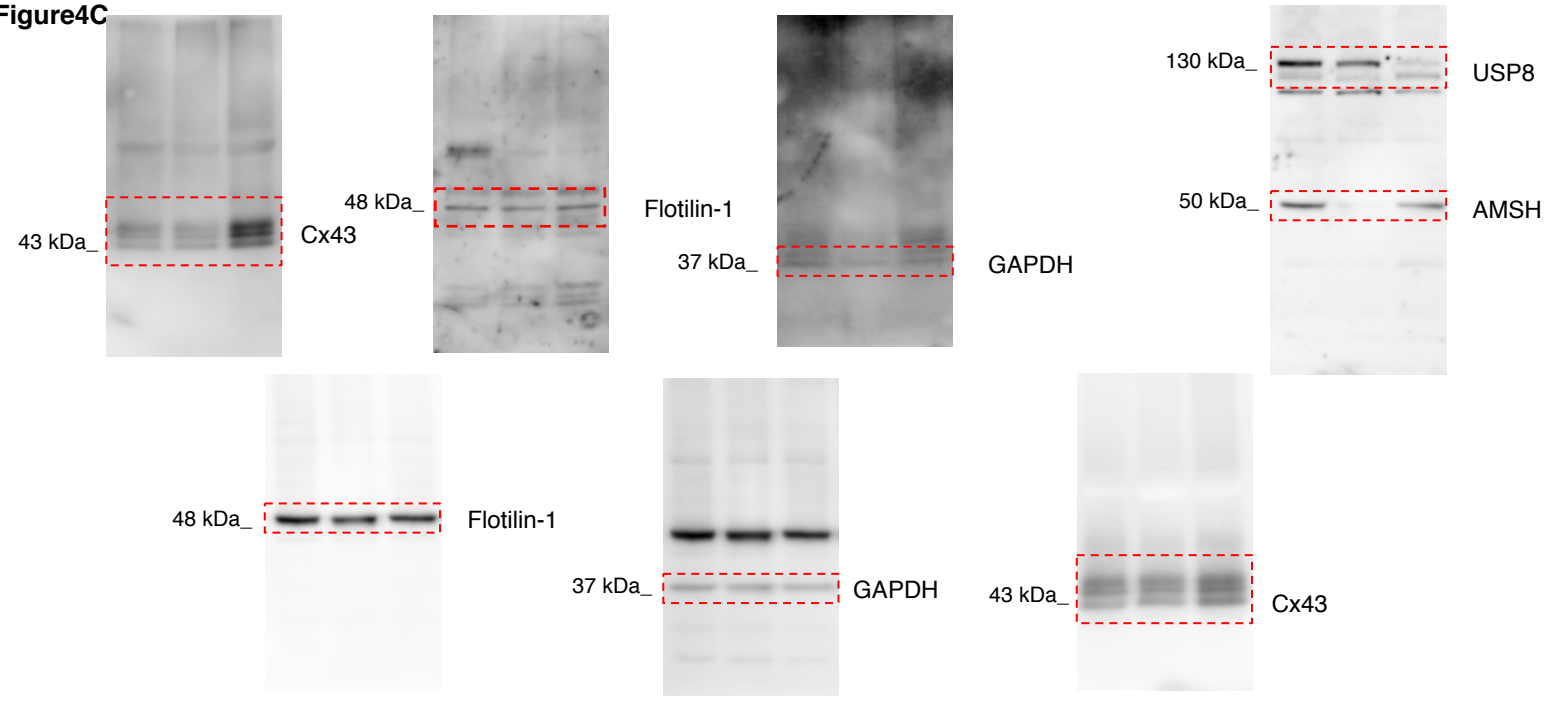

Supplement: Supplementary file 4 [file LSA-2020-00821_SdataF4.pdf]

**Figure5A**

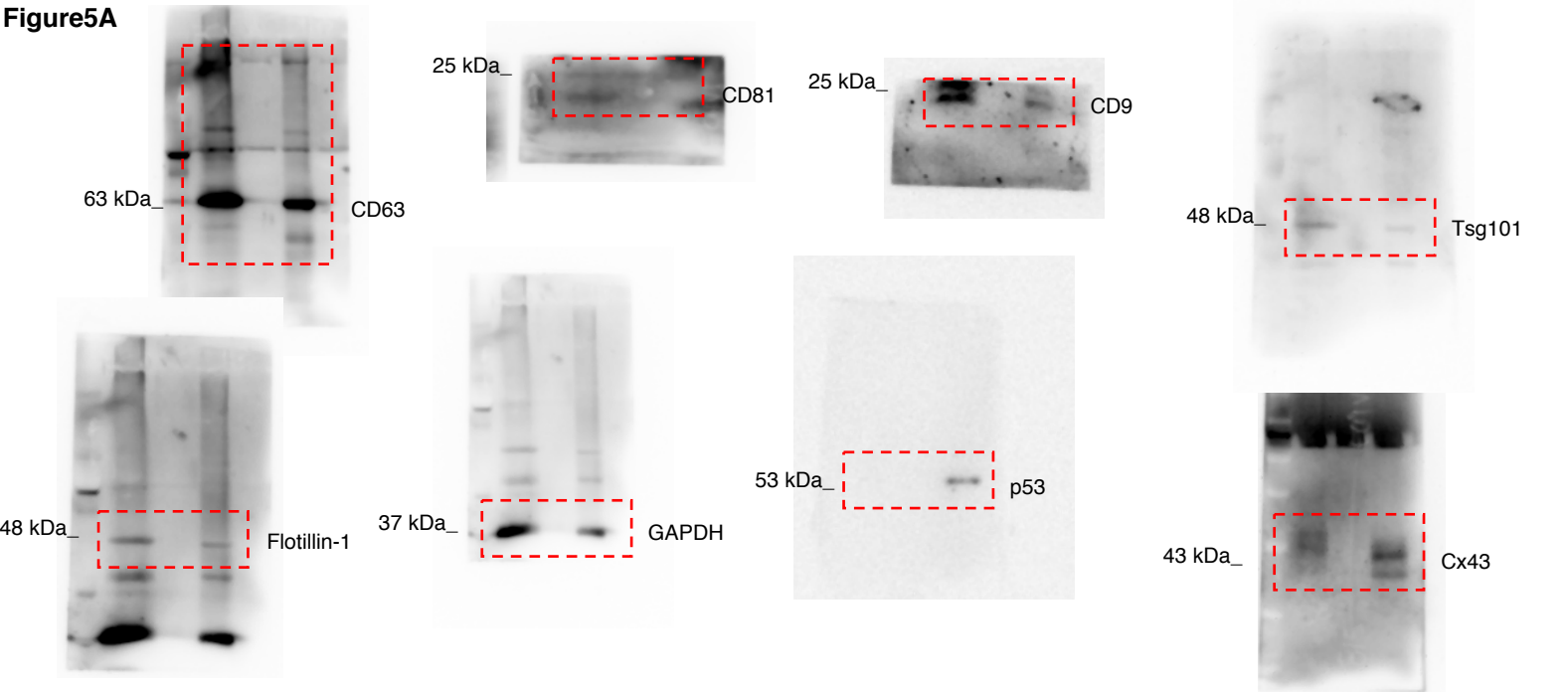

**Figure5C**

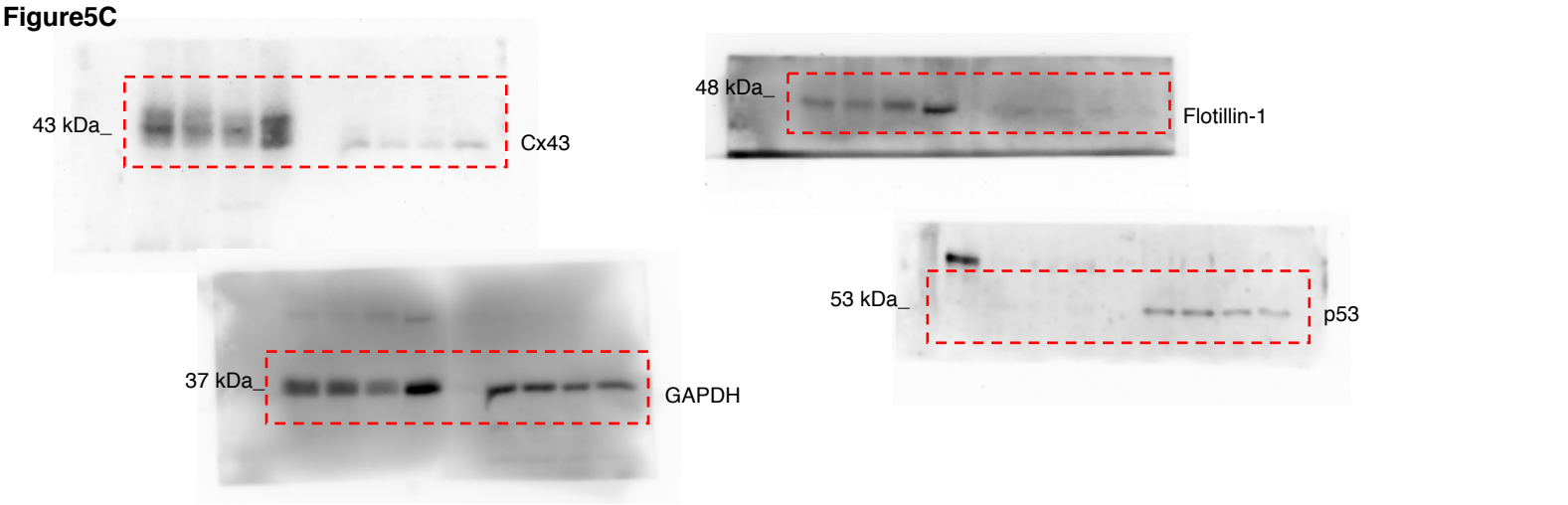

**Figure5D**

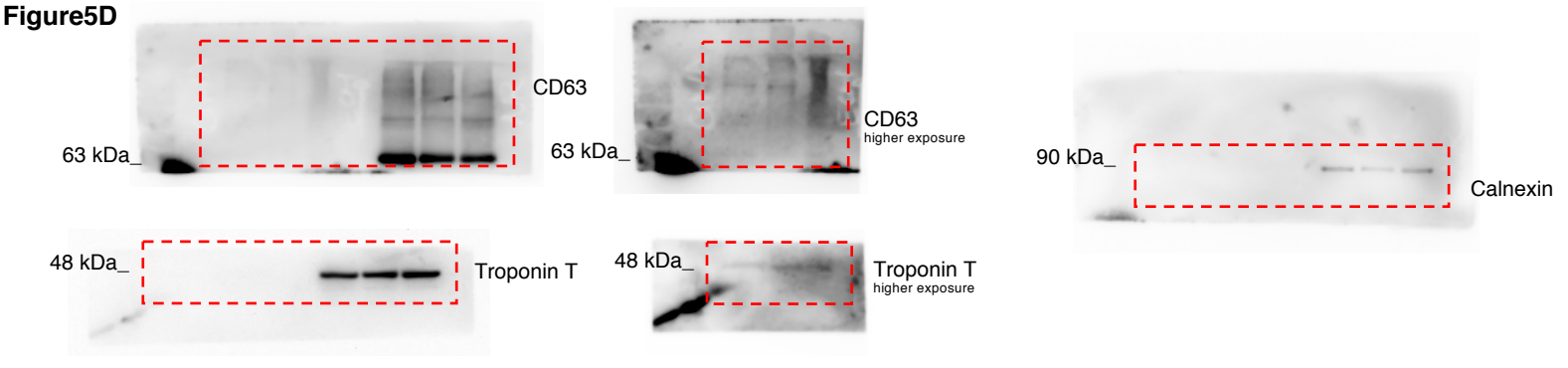

**Figure5E**

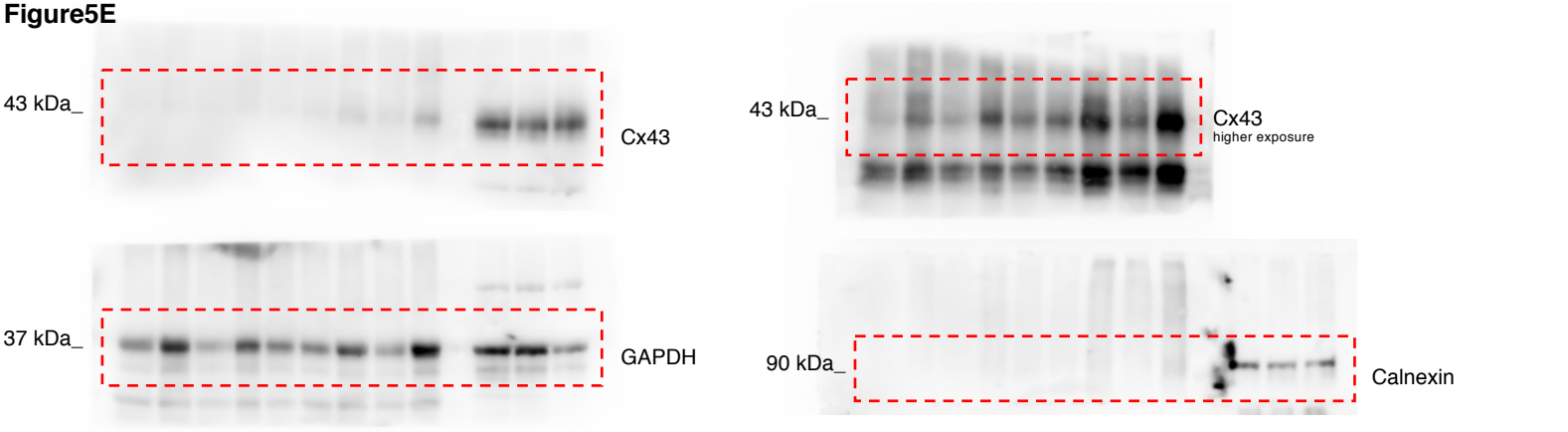

Supplement: Supplementary file 5 [file LSA-2020-00821_SdataF5.pdf]

**Figure6A**

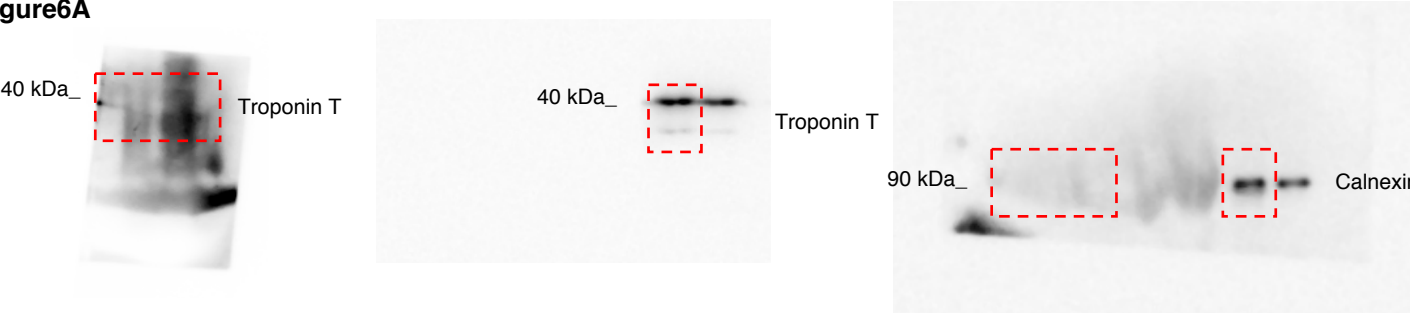

**Figure6D**

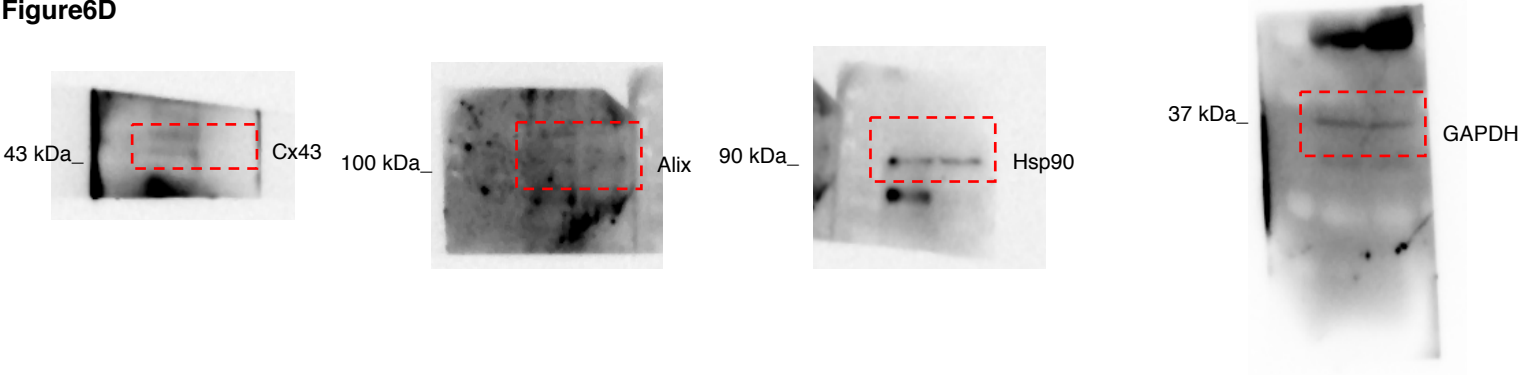

**Figure6E**

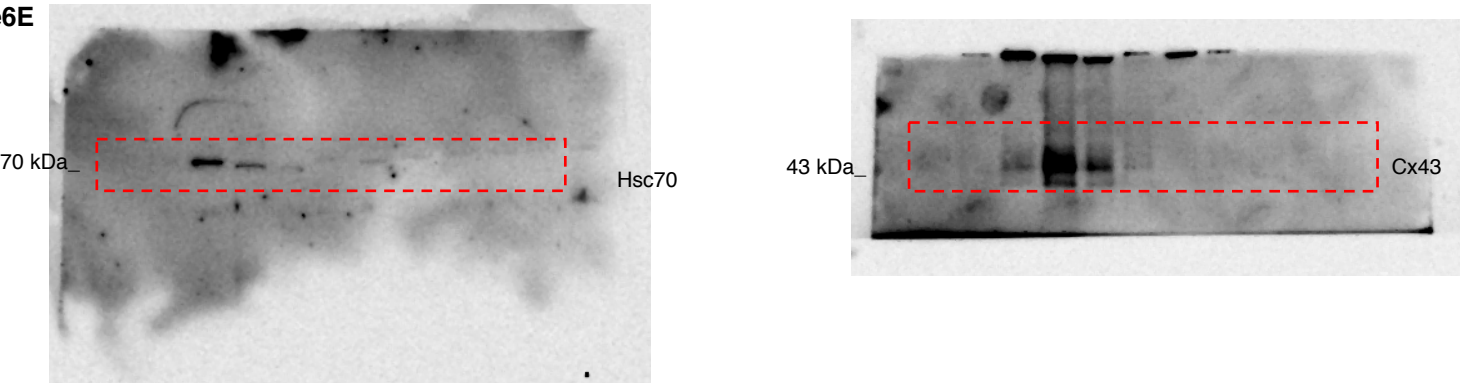

Supplement: Supplementary file 6 [file LSA-2020-00821_SdataF6.pdf]
